# Evidence That 2n Eggs Explain Partial Hybrids between *Medicago sativa* and *Medicago arborea*

**DOI:** 10.3390/plants11101380

**Published:** 2022-05-23

**Authors:** Edwin Bingham, John Irwin

**Affiliations:** 1 Agronomy Department, University of Wisconsin, Madison, WI 53706, USA; 2 School of Agriculture and Food Science, University of Queensland, Brisbane, QLD 4072, Australia; j.irwin@uq.edu.au

**Keywords:** species, subspecies, interspecific hybrids, unreduced gametes, genome, hybrid breakdown, plant breeding

## Abstract

Selected genotypes of alfalfa (*Medicago sativa*) produce partial hybrids in sexual crosses with *Medicago arborea*, as reported in *Plants* (2013). The hybrids contain mostly alfalfa DNA and traits, but also contain DNA and traits from *M. arborea*. It was proposed in 2008 that the partial hybrids could be explained by fertilization of 2n eggs in alfalfa by normal pollen from *M. arborea*, followed by partial loss of *M. arborea* chromosomes during embryogenesis. In this paper, we confirm the presence of 2n eggs in the first alfalfa parents that produced hybrids. The test for 2n eggs involved pollinating 4x alfalfa with pollen from 8x alfalfa. The production of 8x progeny in the cross proved that selected alfalfa parents produced 2n eggs. Thus, 2n eggs appear to explain how the partial hybrids (hereafter hybrids) contain mostly alfalfa DNA and traits. However, two of the six alfalfa plants that did not hybridize with *M. arborea* also had 2n eggs. Thus, although 2n eggs explain the alfalfa content of hybrids, 2n eggs are not the only factor involved in weakening the hybridization barrier, and in transferring genes to alfalfa from *M. arborea*.

## 1. Introduction

Herbaceous alfalfa, *Medicago sativa* (L.) and woody *M. arborea* (L.) were considered reproductively isolated until 2003, when an alfalfa seed parent was found in Wisconsin that produced hybrids [1]. The interspecific hybridization was repeated in Australia and chromosome counts indicated hybrids were 4x/near 4x [2]. Analysis of DNA indicated that hybrids contained mostly alfalfa DNA and traits, but also contained DNA and traits from *M. arborea*. It was proposed in 2008 that hybrids with mostly alfalfa DNA and traits could be explained by hybridization of 2n eggs in alfalfa with normal pollen from *M. arborea*, followed by loss of *M. arborea* chromosomes during embryogenesis [2]. In 2013, we reported that the hybridization barrier was weakened by selection of parents [1]. Since that time, genes have been transferred from woody *M. arborea* to alfalfa several times in sexual crosses. Over 60 hybrids have been produced and intercrossed to produce a cultigen named Alborea [3]. Alborea plants are cross fertile with each other and with alfalfa. Alborea is being used to restructure alfalfa by transferring traits to alfalfa from *M. arborea* [3].

Crosses of *M. arborea* X alfalfa produced no hybrids [1], thus the hybridization barrier is intact in *M. arborea*. The barrier also is intact in most alfalfa plants, as reported in 2013 [1]. Thus, there must be factors in the selected alfalfa parents that permit hybridization. Several seed production factors were tested and ruled out in the 2013 study. These included pollen mentoring, self-seed competition, and pod retention, but information on 2n eggs was not available. Since that time, a test for two eggs was done by crossing 4x alfalfa X 8x alfalfa. Octoploid alfalfa pollen parents were grown from remnant seed of earlier studies [4,5]. Production of 2n eggs in the present study was confirmed by 8x progeny from the cross, as herein reported.

## 2. Results

### 2.1. Pollen Size in the 2n Egg Test

**Figure 1:**
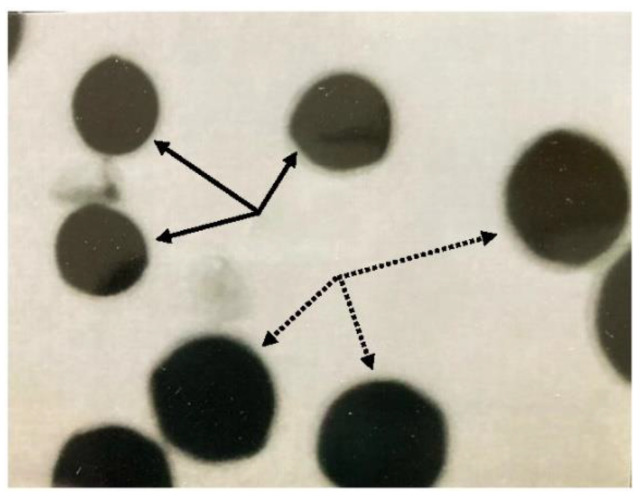
Smaller n = 2x pollen grains (solid arrows) typical of tetraploid pollen, and larger n = 4x grains typical of octoploid pollen (dotted arrows). Photo X450; 1 cm = 40 microns.

Pollen size was used to confirm 8x progeny in the 2n egg test. The pollen in Figure 1 illustrates the size difference between pollen from 4x and 8x plants. Pollen from 4x plants averages 47 microns. Pollen from 8x plants is about one and one-half times larger and averages 70 microns. Pollen in Figure 1 is from a 4x plant that produced n and 2n pollen. The larger 2n pollen is the same size as n = 4x pollen from an 8x plant, and in this case, the two sizes are in the same genetic background. The relationship between pollen size and chromosome number has been recently reviewed [6], and reported in alfalfa studies [5,7,8,9].

### 2.2. Identification of Plants That Produce 2n Eggs

The 2n egg test (see Materials and Methods) is based on a 4x X 8x cross, and identifies 4x plants that produce 2n eggs when they produce 8x progeny (Table 1). The 4x X 8x cross produces mainly 6x embryos, most of which abort. Thus, little or no seed is produced on male sterile plants if they produce only normal n eggs. However, if male sterile plants produce both n and 2n eggs, the frequency of 2n eggs is determined by the number of 8x progeny. In general, the frequency of 2n eggs is low in most plants (Table 1). Male sterile and self-sterile plants have a small amount of pollen, and usually also produce some self-progeny, along with 8x progeny if they have 2n eggs.

Alfalfa seed parents tested for 2n eggs are reported in Table 1. Alfalfa plants MBms and M8 were the first plants that produced hybrids with *M. arborea* [1], and are in bold lettering in Table 1. In the 4x X 8x crosses, both plants produced 8x progeny when crossed with octoploid alfalfa. Thus, both produce 2n eggs along with normal n eggs, since they are fully fertile in crosses with alfalfa. Additional alfalfa parents tested 6-4ms, SX-1, 2K-1ms-1, 2K-1ms-2, and 2K-1ms-3 were descended from male sterile plants that had been crossed with *M. arborea* over the years, but never produced a hybrid [1]. Of these, 2K-1ms-1 and 2K-1ms-3 produced 8x progeny, indicating that they produce 2n eggs, while the others did not. A plant from the Australian cultivar Pegasis was included, and it did not have 2n eggs. Thus, four out of eight alfalfa plants produced 2n eggs in a relatively small number of test crosses.

Male sterile plants 2K-1ms-1, MBms, and self-sterile M8 each produced 2–3 progeny that were 4x (Table 1). In all cases, the 4x progeny had maternal flower color, and were likely self-progeny. The small number of self-progeny indicates self-pollen grains were rare.

## 3. Discussion

The alfalfa parents that produced hybrids in crosses with *M*. *arborea* in earlier studies [1] were found to produce 2n eggs in this study. This confirms the proposition by Armour et al. in 2008 [2] that the hybrids with mostly alfalfa DNA could be explained by union of 2n eggs in alfalfa X n pollen from *M. arborea*. Two alfalfa plants produced 2n eggs, but did not produce hybrids in crosses with *M. arborea.* Thus, 2n egg production explains the alfalfa contribution to the hybrids, but indicates that there are additional factors involved in the hybridization barrier. Several seed production factors were examined but ruled out in our earlier study [1]. Mentoring of *M. arborea* pollen by occasional pollen produced by male sterile alfalfa was ruled out, as was retention of seed pods when a normal seed developed with a hybrid seed. Recently, seasonal effects also were ruled out.

The 4x X 8x cross used to test for 2n eggs produces mainly 6x embryos, most of which abort due to endosperm failure caused by unbalanced fertilization of the endosperm mother cell [10]. Hexaploid embryos sometimes survive [7], but at a lower frequency than expected in the present study where only 50–100 florets were pollinated.

The fact that four out of eight alfalfa plants in this study produced 2n eggs suggests that this abnormality is relatively common in alfalfa. Moreover, this abnormality was found after making a relatively small number of crosses. A higher number of crosses would likely find more. Alfalfa is known to have abnormalities in meiosis [5,7,8,9], and in a study involving 20 alfalfa plants, every plant had an abnormality in some stage of meiosis [11]. Weakening of the hybridization barrier is likely associated with reproductive abnormalities in alfalfa.

Interspecific hybrids that contain mostly the crop plant traits and a fraction of the alien species, as in the alfalfa hybrids, have been found in Brassica, maize, rice, and several other crops. In reviews of these crops [12,13], the crop plant component of the unbalanced hybrids was sometimes attributed to chromosome doubling, or to unreduced 2n gametes. In alfalfa, the production of 2n eggs in the parents of hybrids has been confirmed.

## 4. Materials and Methods

### 4.1. Plant Materials

Plants were maintained as clones periodically propagated by shoot cuttings. Alfalfa and seed parents were from breeding populations adapted to Wisconsin, except for Pegasis from Australia. Octoploid pollen parents were from earlier experiments [4,5]. Octoploids with 2n = 8x = 64 chromosomes make mistakes in both mitosis and meiosis, requiring selection of plants with relatively uniform large pollen, about one and one half times the diameter of tetraploid pollen.

### 4.2. The 2n Egg Test

The test for 2n eggs involved a 4x X 8x cross. The 4x tetraploid alfalfa plants have 2n = 4x = 32 chromosomes. Normal eggs have n = 16 chromosomes, and abnormal 2n eggs have 2n = 32. In the 4x X 8x cross, the union of normal eggs n = 16 X n = 32 normal pollen produces 48 chromosome 6x embryos, most of which abort (see Discussion). Plants test positive for 2n eggs when the union of abnormal eggs 2n = 32 X n = 32 normal pollen produces a 64 chromosome 8x embryo, seed, and plant. Plants are grown to maturity and 8x confirmed by pollen size. 

Alfalfa plants MBms and M8, used to produce the first hybrids, were tested, as were six alfalfa male sterile genotypes that did not produce hybrids. Male sterile alfalfa seed parents were used to minimize self-pollination.

The alfalfa (4x) seed plants were crossed by hand in the greenhouse in winter in Wisconsin. Three to four racemes, each with 12–15 open florets, were pollinated with pollen from 8x plants (reported as 50 florets in Table 1).

Seed produced in the 2n egg test was sown in peat moss-based potting media, and plants grown to flowering for confirmation of octoploids by pollen size. *Medicago* pollen is oblong, sticky and clumpy, but quickly becomes spherical and easy to measure in water and most stains. Pollen was placed on a slide in a drop of aniline blue in lactophenol, and the pollen observed under low power (×100) on a light microscope. Octoploid progeny were confirmed by pollen size; the relationship between size and chromosome number having been established in alfalfa [5,7,8,9].

## 5. Conclusions

Alfalfa plants that produced partial hybrids in crosses with *M. arborea* were found to produce 2n eggs. This supports the suggestion in 2008 that the alfalfa content in the hybrids could be explained by 2n eggs that were fertilized by pollen from *M. arborea*, followed by partial loss of *M. arborea* chromosomes. Some plants that did not produce hybrids with *M. arborea* also produced 2n eggs. Thus, the alfalfa content of hybrids can be explained by 2n eggs, but 2n eggs are not the only factor enabling the interspecific hybridization. Additional reproductive factors appear involved in the weakening of the hybridization barrier.

## Figures and Tables

**Table 1:** Alfalfa seed parents tested for 2n eggs with pollen from 8x alfalfa; a 4x X 8x cross. Bold lettering indicates plants that produced hybrids with *M. arborea* in an earlier study [1].

Plants	Description and Pedigree	Seed */Florets Pollinated	Progeny	
			8x/Near 8x	4x
6-4ms	Male sterile plant discovered in cv. Saranac.	0/100	-	-
SX-1ms	Male sterile plant from a breeding population.	0/50	-	-
2K-1ms-1	*Ibidem*	6/100	2	3
2K-1ms-2	*Ibidem*	0/50	-	-
2K-1ms-3	*Ibidem*	3/50	2	-
Pegasis	Self-sterile plant found in Australian cultivar.	2/50	-	-
**MBms**	Male sterile from Magnum X Blaser XL cross [1].	7/100	5	2
**M8**	Self-sterile from cross of *Medicago* subspecies [1].	12/100	9	2

* One or more seeds or seedlings not viable.
